# Daptomycin Resistance in Clinical MRSA Strains Is Associated with a High Biological Fitness Cost

**DOI:** 10.3389/fmicb.2017.02303

**Published:** 2017-12-05

**Authors:** Melanie Roch, Paula Gagetti, James Davis, Paola Ceriana, Laura Errecalde, Alejandra Corso, Adriana E. Rosato

**Affiliations:** ^1^Department of Pathology and Genomic Medicine, Center for Molecular and Translational Human Infectious Diseases Research, Houston Methodist Research Institute, Houston, TX, United States; ^2^Servicio Antimicrobianos, Instituto Nacional de Enfermedades Infecciosas, Administración Nacional de Laboratorios e Institutos de Salud, Buenos Aires, Argentina; ^3^Argonne National Laboratory (DOE), Lemont, IL, United States; ^4^Computation Institute, University of Chicago, Chicago, IL, United States; ^5^Departamento de Microbiologia, Hospital General de Agudos Juan Fernandez, Buenos Aires, Argentina

**Keywords:** MRSA, daptomycin, biological cost, *in vivo*

## Abstract

Daptomycin remains as one of the main treatment options for Methicillin-Resistant Staphylococcus aureus (MRSA). Sporadic resistance cases reported in patients treated with either daptomycin or glycopeptides are a growing concern. In a previous study, we described a clinical case of a patient with a community-acquired MRSA infection resistant to daptomycin and with intermediate resistance to vancomycin who developed a recurrent infection with a susceptible isogenic strain. In the present work, we further investigated the sequential events to determine whether the switch from a daptomycin resistance to a susceptible phenotype was due to a phenomenon of resistance reversion or recurrent infection with a susceptible strain. Pairwise competition experiments showed that the susceptible clinical recurrent SA6850 strain had increased fitness when compared to the resistant counterpart SA6820 strain. In fact, although we have demonstrated that reversion of daptomycin resistance to daptomycin susceptible can occur *in vitro* after serial passages in drug-free media, phylogenetic analysis suggested that the *in vivo* process was the result of a recurrent infection with a previous susceptible isolate carried by the patient rather than a resistance reversion of the strain. Whole genome sequence of evolved strains showed that daptomycin resistance in MRSA is associated with a high fitness cost mediated by mutations in *mprF* gene, revealed as a key element of the biological cost. Moreover, we determined that daptomycin resistance-associated fitness cost was independent of vancomycin intermediate resistance phenotype, as demonstrated in additional clinical MRSA vancomycin susceptible strains. This study highlights important observations as, despite daptomycin offers a useful treatment option for the patients with persistent infections, it has to be carefully monitored. The high fitness cost associated to daptomycin resistance may explain the reduced dissemination of daptomycin resistance and the absence of daptomycin reported outbreaks.

## Introduction

*Staphylococcus aureus* is one of the most common human pathogens isolated from both community-acquired (CA) and hospital-acquired (HA) infections. Antibiotic management of those infections has become complex over the last decades due to large spreads of multiresistant strains, notably methicillin resistant *S. aureus* (MRSA) and vancomycin intermediate *S. aureus* (VISA) (Stryjewski and Corey, 2014). Daptomycin (DAP), a calcium-dependent lipopeptide antibiotic with potent bactericidal activity (Kanafani and Corey, 2007), is the main alternative for the treatment of MRSA infections in cases of vancomycin-reduced susceptibility or vancomycin treatment failure (Liu et al., 2011). The mechanism of action involves the functional disruption of the cytoplasmic membrane which leads to its depolarization and cell death (Bayer et al., 2013). *S. aureus* strains with minimal inhibitory concentration (MIC) ≤ 1 mg/L are classified as DAP susceptible (DAP-S) according to Clinical and Standard Laboratory Institute (CLSI, 2015). As no resistant breakpoint has been officially established, strains with MIC > 1 mg/L should be referred to as non-susceptible. However, the term resistant (DAP-R) will be used in the present study to simplify the understanding.

Acquisition of DAP resistance in *S. aureus* is a stepwise and multifactorial process that includes cell membrane and probably cell wall perturbations (Baek et al., 2015; Tran et al., 2015). Mutations have been described in various genes including those associated with the cell membrane (*mprF*), cell wall (*walKR*, *dltABCD*, and *yycG*), and RNA polymerase subunits (*rpoC* and *rpoB*), both in clinical isolates and laboratory derivatives (Friedman et al., 2006; Cui et al., 2010; Mehta et al., 2011; Mishra et al., 2014; Baek et al., 2015). Moreover, the two-component system VraSR, a key regulator of the cell wall synthesis, has also been shown to play an important role in DAP resistance regulation (Mehta et al., 2011). The gene most consistently associated with the resistance in clinical and *in vitro* mutant strains is *mprF*, previously known as *fmtC* (Peleg et al., 2012). The transmembrane protein MprF is responsible for the lysinylation of cell membrane phospholipids and their translocation to the outer leaflet of the cell membrane (Ernst et al., 2009). Gain-in-function mutations of *mprF* increase the cell membrane positive charges, and thus repulse the calcium-complexed DAP (Jones et al., 2008; Bayer et al., 2013).

Although the development of DAP resistance remains rare, cases have been reported in patients treated for high bacterial inoculum infections under antimicrobial therapies with glycopeptides or DAP, resulting in treatment failure, poor clinical outcomes, and high mortality (van Hal et al., 2011; Capone et al., 2016).

In a previous report, we described a clinical case of a patient in Argentina with a CA-MRSA infection with intermediate resistance to vancomycin (VISA) and resistance to DAP who developed a recurrence with a susceptible isogenic strain (Errecalde et al., 2013). A similar case was reported in 2016 by Capone et al. (2016) where under DAP treatment, a MRSA isolate acquired a heterogeneous DAP resistance that, surprisingly, came back to a susceptible phenotype when the treatment was switched. Both cases described a putative reversion of DAP resistance; however, the bases of this process remained to be determined.

In the present investigation, by using competition and phylogenetic analyses of the successive isolates originated from the same patient, we show that DAP resistance in MRSA is associated with a high fitness cost mediated by mutations in *mprF* gene, and that *in vivo* conversion of DAP resistance to a susceptible phenotype was in fact a recurrent infection rather than reversion.

## Materials and Methods

### Bacterial Strains

Consecutive CA-MRSA clinical isolates (ST5-SCC*mec* type IV-PVL+) were recovered from blood cultures from a patient with chronic kidney failure under dialysis with a history of fracture osteosynthesis: SA6819 (h-VISA/S-DAP), SA6820 (VISA/R-DAP), and SA6850 (VSSA/S-DAP) (Errecalde et al., 2013). The SA6820Δ*mprF* was obtained by transduction from the strain CK1001 using phage 80α. The clinical isogenic pairs DAP-S and DAP-R CB-1631(S)/CB-1634(R) and CB-5013(S)/CB-5014(R) (Mehta et al., 2011) described in **Table 1** were used as representative strains displaying DAP-R and vancomycin susceptible phenotype (VSSA) for studies of DAP resistance fitness cost in vancomycin susceptible isolates.

**Table 1:** Description of the strains used in this study and their MICs obtained by *E*-test^®^.

Strain	MIC DAP (mg/L)	MIC VAN (mg/L)	Source and reference
SA6819	0.25	2	Clinical strain, Errecalde et al., 2013
SA6820	4	4	Clinical strain, Errecalde et al., 2013
SA6850	0.25	1	Clinical strain, Errecalde et al., 2013
SA6820-revertant D35	0.25	2	Laboratory derived, this study
SA6820 Δ*mprF*	0.75	3	Laboratory derived, this study
CB-1631	0.5	1	Clinical strain, Cubist Pharmaceutical, Mehta et al., 2011
CB-1634	4	2	Clinical strain, Cubist Pharmaceutical
CB-5013	0.5	1	Clinical strain, Cubist Pharmaceutical
CB-5014	4	2	Clinical strain, Cubist Pharmaceutical

### Antibiotics and Susceptibility Testing

Daptomycin was provided by former Cubist/Merck Pharmaceuticals. Susceptibilities to vancomycin and DAP were performed by *E*-test (BioMérieux, Marcy l’Etoile, France). Antimicrobial susceptibility testing was performed by Kirby–Bauer method and according to the guidelines of the Clinical and Laboratory Standards with antimicrobial Sensi-disks (BD, Franklin Lakes, NJ, United States). The antimicrobial susceptible testing was used to complement genetic approaches to evaluate the antibiotic susceptible phenotype related to the occurrence of specific mutations and to follow up the identity of the strains during long-term passages experiments. The antibiotics tested were rifampicin (RIF), vancomycin (VAN), ciprofloxacin (CIP), and chloramphenicol (CM).

### Illumina Short Read Genome Sequencing

Genomic DNA of all strains was extracted from an overnight culture grown in BHI at 37°C using the DNeasy Blood and Tissue Kit (QIAGEN, Hilden, Germany). Libraries were prepared from purified DNA using Nextera XT DNA Library Preparation Kit (Illumina, San Diego, CA, United States) and sequenced (50 nucleotide reads) with HiSeq 2000 instruments at the Epigenetics and Genomic Laboratory at Weill Cornell University, New York, NY, United States.

### Templated Genome Assembly

Templated assemblies of the short reads were performed versus N315 (GenBank accession number BA000018) and the SNPs identified using the Lasergene 13 Suite Software (DNASTAR, Inc., Madison, WI, United States).

### Sanger DNA Sequencing

The *mprF* mutations were confirmed by PCR and Sanger sequencing using previously described *mprF* primers (Mehta et al., 2011) at the Nucleic Acid Research Facility at Genewiz (South Plainfield, NJ, United States).

### Phylogenetic Analysis

All *S. aureus* strains of the ST5 MLST type were downloaded from PATRIC in April, 2017 (Wattam et al., 2017). Strains with a BioSample identifier that unambiguously linked to a single run accession were downloaded from the European Nucleotide Archive (Leinonen et al., 2011). When read files were paired-end, only one set in the pair was used for the comparison. Reads were aligned against N315 (PATRIC ID: 158879.11) and analyzed using the PATRIC variation service (Wattam et al., 2017), which uses BWA-MEM as the read aligner^1^ and FreeBayes as the SNP caller^2^. A SNP alignment was generated by using a method previously described (Leekitcharoenphon et al., 2012). A tree for the alignment was generated using FastTree. Genes for the RNA polymerase beta-prime subunit (*rpoC*) were downloaded from PATRIC in April, 2017. Genes were aligned using MAFFT and a tree was built using FastTree. Trees were rendered using the iTOL website (Letunic and Bork, 2007).

### *In Vitro* DAP Resistance Reversion

To access the ability of DAP resistance to revert, the resistant strain SA6820 was serially passaged daily in drug-free BHI broth. Every 5 days, enumeration of the DAP-resistant population was performed by plating the culture and its serial dilutions on BHI agar supplemented with calcium (CaCl_2_, 50 mg/L) and containing or not DAP 2 mg/L. The mutational genetic events of the resulting strain obtained after 35 days (SA6820-revertant) were determined by whole genome sequencing and its fitness was compared against its parental SA6820 strain.

### Competition Experiments and Single Cultures

Pairwise competition experiments between isogenic strains were conducted to compare their fitness (Andersson and Hughes, 2010). This method enables a good discrimination by taking into account the different component of the fitness, including the lag period and the exponential growth phase. Briefly, overnight cultures of the strains were mixed in equal proportion and inoculated at 10^5^ CFU/mL in 10 mL of BHI broth (BD, Franklin Lakes, NJ, United States). The mixed 24-h culture was then diluted 1:1000 in fresh BHI broth and repeated daily during 10 days. Enumeration of the DAP resistant population was performed every 2 days followed by plating the culture and its serial dilutions on BHI agar supplemented with calcium (CaCl_2_, 50 mg/L) and containing or not DAP 2 mg/L. The final level of DAP resistance of the population was also evaluated after the 10 days of competition by *E*-test (BioMérieux, Marcy l’Etoile, France) with a 0.5 McF inoculum and directly from the whole population cells. Single cultures of the resistant strain SA6820 were conducted in parallel of the competition assay by daily passage in drug-free BHI broth to assess their stability during the competition experiment. The susceptible strain SA6850 was also plated on BHI containing DAP to evaluate the spontaneous mutation frequency. For the competitive culture between SA6850 and SA6820*ΔmprF* that are both DAP-S, respectively; CIP 2 mg/L and CM 10 mg/L were used as antimicrobial markers to identify SA6850 (CIP-R) and for SA6820Δ*mprF* (CM-R).

## Results and Discussion

### *mprF* Is Associated with DAP Resistance in the ST5-SCC*mec* Type IV-PVL+ SA6820 Isolate

**FIGURE 1:**
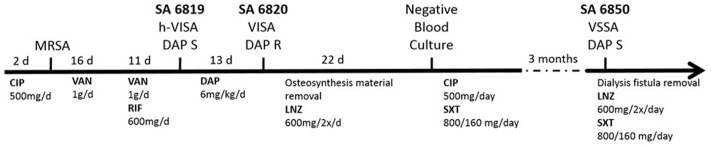
Timeline of the infection and treatments related to the isolation of the clinical SA6819, SA6820, and SA6850 strains. (d: day, CIP: ciprofloxacin, DAP: daptomycin, LNZ: linezolid, RIF: rifampicin, SXT: trimethoprim/sulfamethoxazole, and VAN: vancomycin).

The patient described in our previous study presented multiple comorbidities (diabetes, chronic kidney failure on hemodialysis, and hip fracture osteosynthesis), and was treated for a persistent bacteremia by various combination of antibiotics including VAN and DAP (van Hal et al., 2011; Bassetti et al., 2015; **Figure 1**). DAP resistance in SA6820 developed concomitantly with the acquisition of a full VISA resistance phenotype (isolate SA6820; VAN: 4 μg/ml) after DAP treatment of a heterogeneous-vancomycin intermediate resistant (h-VISA: 2 μg/ml) isolate (SA6819) (**Figure 1**). Whole genome sequencing analysis revealed that SA6820 carried the L826F *mprF* mutation, one of the most commonly found in DAP resistant isolates (Bayer et al., 2015) and shown to be a major determinant of DAP resistance (Mehta et al., 2011; Yang et al., 2013). Additional SNPs in SA6820 were present in *rpoB* gene at position A477D, and *vraT* at position H3_S7delinsP (**Table 2**). After treatment was switched to LNZ (linezolid), CIP and SXT (trimethoprim/sulfamethoxazole) and following a 3-month period of clinical improvement, the patient developed a recurrent infection due to a DAP susceptible isolate (SA6850).

**Table 2:** Most relevant mutations related to daptomycin and vancomycin resistance carried by the strains.

Strain	*rpoB*	*mprF*	*yycG* (walk, *vicK*)	*vraS*	*vraT* (*yvqF* /SA1702)	DAP	VAN	RIF	CIP
SA6819	H481Y	–	–	–	–	S	S	R	S
SA6820	A477D	L826F	–	–	H3_S7delinsP	R	I	R	S
SA6850	H481Y	–	–	–	–	S	S	R	R
SA6820-revertant	A477D D471A	Y116fs^∗^	–	E316V	H3_S7delinsP	S	S	R	S
SA6820 Δ*mprF*	A477D	Δ*mprF*	–	–	H3_S7delinsP	S	I	R	S
CB-1631	–	–	–	–	–	S	S	S	R
CB-1634	–	L826F	K159fs	–	–	R	S	S	R
CB-5013	–	–	–	–	–	S	S	S	R
CB-5014	–	S337L	–	–	–	R	S	S	R

^∗^*The L826F mutation was still present but will not be translated due to the reading frameshift leading to an early stop codon in position 126*.

However, sequence analysis of SA6850 displayed a mutation in the *rpoB* gene at position H481Y similar to the susceptible SA6819, while mutations in *mprF* and *vraT* were no longer present. The changes in genotype in SA6850 were consistent with its susceptible phenotype to DAP and VAN. The three isolates were shown to be classified as the same clone by PFGE and MLST (Errecalde et al., 2013) corresponding to ST5-SCC*mec* type IV-PVL+, one of the main community-acquired clones circulating in Argentina; the high homology between these strains was confirmed by the phylogenetic tree based on *rpoC* (Supplementary Figure S1). These results were consistent with two hypotheses: (i) the susceptible SA6850 strain could be an *in vivo* revertant of the resistant SA6820 or (ii) it could be a recurrence of a previous version of the strain that the patient carried before acquisition of the DAP resistance. In both proposed hypotheses, the underlying condition was that the resistant SA6820 strain had a fitness disadvantage compared to SA6850, which allowed the replacement of the resistant strain by a susceptible one.

### Strain SA6850 Outgrew SA6820 because of Its Better Fitness

**FIGURE 2:**
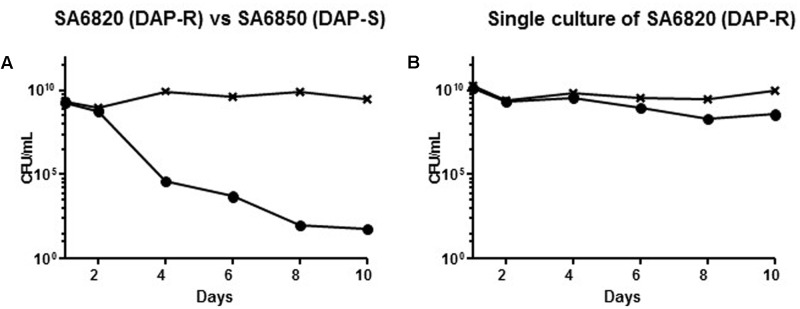
**(A)** Competition experiment between the clinical SA6820 (DAP-R) and the clinical recurrent SA6850 (DAP-S) strains. The DAP-R sub-population of SA6820 is indicated with (•), the whole population (SA6820 + SA6850) growing on BHI agar without antibiotic is indicated with (**x**). **(B)** Single culture of SA6820 showed that in absence of competition with a susceptible strain, the resistance is stable during 10 days.

To test the fitness difference, we performed *in vitro* competitive cultures between DAP resistant SA6820 and its susceptible counterpart SA6850. After 10 days of subculture, the DAP susceptible strain SA6850 had completely outgrown the DAP resistant SA6820 (**Figure 2A**). MIC determination performed by *E*-test after 10 days of culture with inoculum from whole population (SA6820 + SA6850) revealed a susceptible phenotype with a DAP MIC at 0.38 μg/mL. Residual population growing on BHI containing 2 mg/L DAP was shown to be spontaneous mutants of SA6850, as indicated by their characteristic resistance to CIP which was not carried by SA6820. In parallel, SA6820 resistant strain in single culture was shown to be stable during the 10 days of the experiment with a decrease of resistance of only 1 log10 (**Figure 2B**) and an MIC by *E*-test of 3 μg/mL. The results of *in vitro* competition experiments are in agreement with the observation that the DAP susceptible SA6850 strain had an increased fitness compared to its DAP resistant counterpart SA6820 strain.

### Investigation of the Origin of the DAP Susceptible SA6850

**FIGURE 3:**
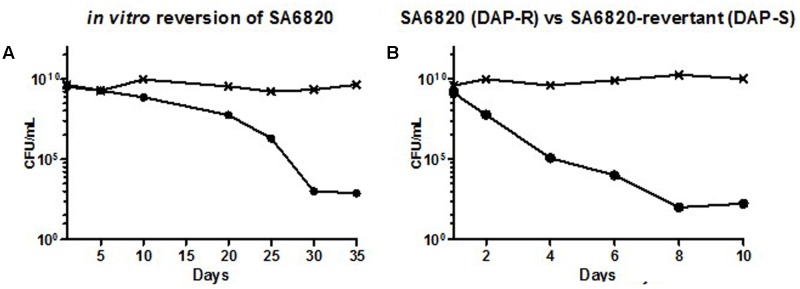
**(A)** Progressive reversion of DAP resistance during extended *in vitro* single culture of the clinical DAP-R strain SA6820 by daily serial passages in drug-free BHI broth. Every 5 days, enumeration of the DAP resistant population was performed by plating the culture and its serial dilutions on BHI agar supplemented with calcium (CaCl_2_, 50 mg/L) and containing or not DAP 2 mg/L resulting in (SA6820-revertant). The DAP-R sub-population of SA6820 is indicated with (•), the whole population (SA6820 + SA6820-revertant) growing on BHI agar without antibiotic is indicated with (**x**). **(B)** Competition experiment between the clinical strain SA6820 (DAP-R) and its *in vitro* revertant SA6820-revertant (DAP-S). The DAP-R sub-population of SA6820 is indicated with (•), the whole population growing on BHI agar without antibiotic is indicated with (**x**).

To address our first hypothesis, i.e., whether DAP resistance may directly revert to a susceptible phenotype, we performed *in vitro* serial passages of SA6820 in drug-free BHI broth to determine the ability of the DAP resistance to revert. We observed a progressive decrease of the DAP resistant population (**Figure 3A**) showing the slow reversion of the resistance. After 35 days, a fully susceptible strain with a DAP MIC at 0.5 μg/mL, named SA6820-revertant, was recovered. A residual population growing on BHI+Ca^2+^ containing 2 mg/L of DAP was present in similar proportion (1.77 × 10^-7^) than the spontaneous DAP resistance frequency previously described in *S. aureus* (Kaatz et al., 2006). The SA6820-revertant also became susceptible to VAN, similarly to the clinical recurrent SA6850 strain.

To determine the *in vitro* generated gene polymorphisms associated with the conversion, the SA6820-revertant strain was full genome sequenced and compared to its parent SA-6820. Interestingly, the revertant strain showed a deletion of a single nucleotide (T) in position 346 leading to a reading frameshift (Y116fs) and an early stop codon in position 126 of MprF, suggesting a direct role of *mprF* in the conversion process and thus in the fitness modification. Additional *de novo* mutations were also found in genes previously described to be associated with vancomycin intermediate resistance (Howden et al., 2010; Hafer et al., 2012) including D471A in RpoB and E316V VraS (**Table 2**), both of which may be also linked to its reversion.

Previous studies have shown that a resistant strain can rapidly acquire compensatory mutations to improve its fitness or adapt its metabolism to its culture media (Andersson and Hughes, 2010). However, in the strain SA6820, spontaneous inactivation of MprF itself occurred, resulting in the restoration of DAP susceptibility. This suggested that MprF inactivation improved the fitness of the strain which progressively outgrew the resistant population leading to a fully susceptible isolate. This result was supported by the competitive culture performed between the SA6820 and its *in vitro* revertant that showed a rapid decrease of the resistant population (**Figure 3B**). The difference of fitness was similar to the one observed between the clinical strains SA6820 and the *in vivo* DAP susceptible recurrent SA6850 strain (**Figure 2B**). Since VAN phenotype appears to be directly affected during the process of reversion and may confound our results, the independent role of DAP resistance was confirmed in additional strains which were DAP resistant but VAN susceptible (these data are shown in separate section of this manuscript).

**FIGURE 4:**
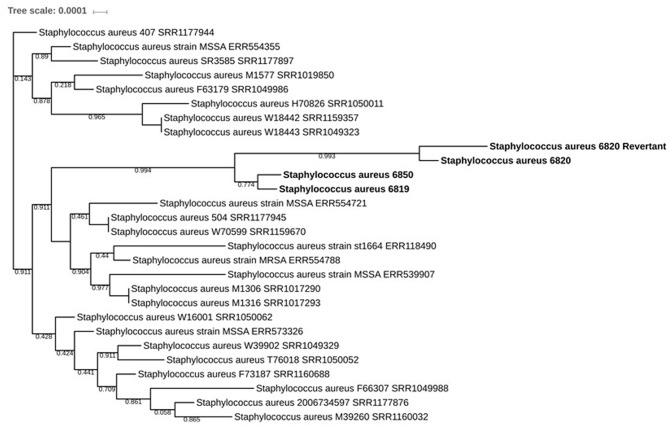
Phylogenetic tree based on the SNP comparisons versus N315 showing the evolution of the different isolates: the strains SA6819, SA6820, SA6820-revertant, and SA6850 are shown in bold. All strains represented are from the ST5 MLST type.

To investigate whether SA6850 was either an *in vivo* revertant or a recurrence of a susceptible strain previously carried by the patient, deeper analysis taking into account all the SNPs identified versus the *S. aureus* N315 reference strain was performed, with the goal of determining the proximity between SA6819, SA6820, SA6850, and the *in vitro* obtained SA6820-revertant strain. The phylogenetic tree revealed that the recurrent susceptible SA6850 strain was closer to the DAP susceptible h-VISA isolate SA6819 than to the VISA DAP resistant SA6820 (**Figure 4**). The high homology between these strains was confirmed by the phylogenetic tree based on *rpoC* (Supplementary Figure S1). These results suggested that the recurrent SA6850 strain was more likely to arise from a competition between a residual DAP susceptible population present in the patient and its environment than from a direct reversion of SA6820 strain. Furthermore, the reversion of DAP resistance to DAP susceptible phenotype observed in our patient was to some extent unexpected because there were no previous reports of cases describing a direct DAP resistance reversion. In fact, a similar clinical case has been then reported in where a heterogeneous MRSA DAP resistance isolate was selected under DAP treatment followed by a susceptible DAP phenotype strain when the antibiotic treatment was changed, however, the mechanistic bases of this phenotypic conversion were not further explored (Capone et al., 2016). Moreover, in a recent clinical MRSA strain pair obtained from a patient, it was demonstrated that DAP resistance reversion to a susceptible phenotype was obtained after DAP discontinuation, mechanistically associated with downregulation of cell wall associated genes (Iwata et al., 2017). These observations including ours, support the common notion that DAP resistant strains have the ability to revert to susceptible once DAP treatment is discontinued and switched to an alternative antimicrobial therapy.

### Impact of *mprF* Mutation on DAP Fitness

**FIGURE 5:**
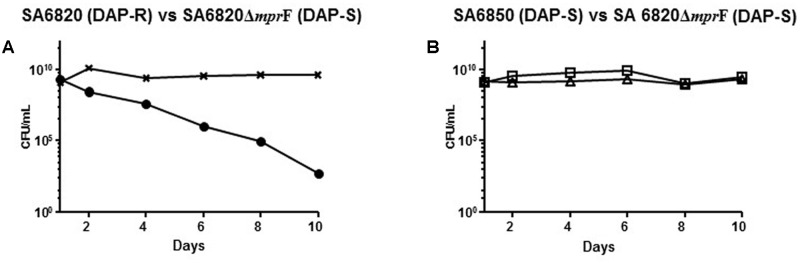
**(A)** Competitive cultures performed between SA6820Δ*mprF* and SA6820 showed increased fitness of SA6820Δ*mprF* over SA6820 (DAP-R). DAP-R sub-population of SA6820 is indicated with (•) and the whole population growing on BHI agar (SA6820 + SA6820Δ*mprF*) is indicated with (**x**). **(B)** Competition experiment between the two DAP-S strains: SA6850 (ciprofloxacin marker □) and SA6820Δ*mprF* (chloramphenicol marker Δ).

The competition experiment between the SA6820 and SA6850 confirmed that the resistant strain had a lower fitness. This observation together with the spontaneous inactivation of MprF during serial passages of the resistant strain suggested that MprF, the major determinant of DAP resistance, may have a key role in the fitness cost of the resistance. To test this hypothesis, *mprF* was inactivated in the DAP resistant strain SA6820 by transduction of an insertional chloramphenicol cassette, resulting in the SA6820Δ*mprF* (CM-R) strain. As expected, this inactivation restored the DAP susceptibility and, of note, also decreased slightly the VAN intermediate resistance (**Table 1**). The *mprF* influence on VAN resistance had been previously suggested by different studies (Ruzin et al., 2003; Chen et al., 2015); however, the mechanism remains unknown. Competitive cultures performed between SA6820Δ*mprF* and SA6820 showed increased fitness of SA6820Δ*mprF* over SA6820 (**Figure 5A**), confirming the impact of *mprF* on the strain fitness. Competition between the two DAP susceptible strains SA6820Δ*mprF* and SA6850 showed no major difference of fitness; as shown in **Figure 5B**, after 10 days both remained in culture in similar proportion as confirmed by their resistance to both ciprofloxacin, characteristic of SA6850, and to chloramphenicol, marker of SA6820Δ*mprF*.

*In vitro* studies proved the ability of DAP resistance to revert within a month (SA6820-revertant D35, DAP MIC: 0.25 μg/mL), a mechanism mediated by the inactivation of *mprF*, the key gene responsible for the resistance. This modification improved the fitness of *mprF*-inactivated population which progressively outcompeted the resistant population. However, we cannot exclude the possibility that the *in vivo* process may differ from the *in vitro* one due to the potential complexity of its environment.

### DAP Resistance Fitness Cost in Vancomycin Susceptible Isolates

**FIGURE 6:**
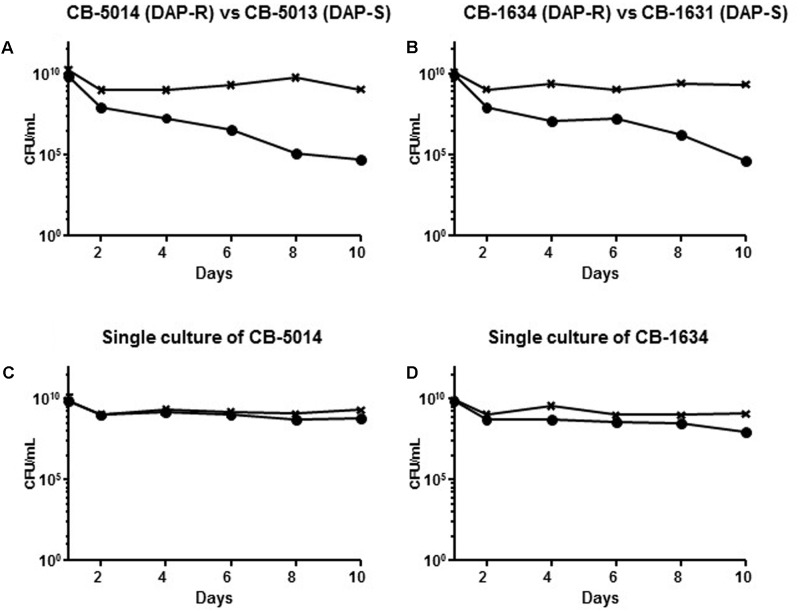
**(A)** Competition experiment between the clinical strains CB-5014 (DAP-R) and CB-5013 (DAP-S) and **(B)** CB-1634 (DAP-R) and CB-1631 (DAP-S) in comparison with the single culture of their corresponding DAP-R resistant counterparts **(C,D)**. The DAP-R sub-population of each experiment is indicated with (•), and is represented by either CB5014 **(C)** or CB1634 **(D)** grown with DAP; the whole population of CB5014 **(C)** + CB1634 **(D)** growing on BHI agar without antibiotic is indicated with (**x**).

The vancomycin intermediate resistance and *rpoB* SNPs carried by SA6820 have been previously associated with a high fitness cost (Boyle-Vavra et al., 2000; O’Neill et al., 2006; Noto et al., 2008), and could potentially confound our results as the strain SA6820 is a VISA strain, as most of the DAP resistant strains (Moise et al., 2009). To test the hypothesis that DAP resistance may have an independent fitness cost, we performed similar competition experiments (DAP susceptible versus resistant) using two pairs of VAN susceptible clinical isogenic strains, CB-5013/CB-5014 and CB-1631/CB-1634. These strains carried different *mprF* mutations, i.e., L826F in the hydrophobic N-terminal translocase, strain CB-1634, and S337L in the central bifunctional domain, strain CB-5014 (Ernst et al., 2009; Bayer et al., 2015). By performing competition analysis, we found that DAP susceptible strains, i.e., CB-5013 and CB-1631, displayed a better fitness as they both outcompeted their corresponding isogenic resistant counterparts (**Figure 6**). These results strengthened our hypothesis showing the *mprF*-mediated DAP resistance fitness cost, and demonstrated that it was independent of the presence of *rpoB* mutations and VAN intermediate resistance.

## Conclusion

In summary, our study demonstrates for the first time the fitness cost of DAP resistance in clinical *S. aureus* and the role of *mprF* in this phenomenon. Therefore, due to the loss of fitness, DAP resistance conversion to DAP susceptibility can occur in patients when antibiotic pressure is removed. Our study demonstrate the possibility that a patient can carry strains with different resistance phenotypes, which can compete with each other and have different selective advantage in different contexts (presence or absence of antibiotics) in this particular case DAP. In fact, although we have demonstrated that reversion of DAP-R to DAP-S can occur *in vitro*, our results revealed that that *in vivo* conversion of DAP resistance to a susceptible phenotype was in fact a recurrent infection rather than reversion as also observed in additional reported cases (Capone et al., 2016; Iwata et al., 2017)

These findings are in support of one of our initial hypotheses in where the resistant SA6820 strain had a fitness disadvantage compared to SA6850, therefore favoring the replacement of the resistant strain by a susceptible one. In fact, MICs should be carefully monitored during the course of persistent infections or recurrence in where DAP may offer a useful treatment option for the patient (Capone et al., 2016), although the probability of resistance re-selection when the antibiotic treatment is re-introduced remains unknown. The difficulty to sustain DAP resistance due to high fitness cost may explain the reduced dissemination of DAP resistance as well as the absence of DAP reported outbreaks. However, our study does not rule out that DAP transmission may occur in the presence of high antibiotic pressure, such as that existing in an intensive care unit where small outbreaks of vancomycin intermediate resistance have been previously reported (Pina et al., 2000; de Lassence et al., 2006).

## Author Contributions

MR: conceived and designed experiments. JD: performed genome and phylogeny analysis. PG: performed data analysis. MR, PG, and AR: drafted the manuscript. LE, PC, and AC: provided clinical strains and performed data analysis. AR, MR, and JD edited the manuscript and did final preparation.

## Conflict of Interest Statement

The authors declare that the research was conducted in the absence of any commercial or financial relationships that could be construed as a potential conflict of interest.

## Footnotes

1 https://arxiv.org/abs/1303.3997

2 https://arxiv.org/abs/1207.3907
