# Supplementary figures and images for: Daptomycin Resistance in Clinical MRSA Strains Is Associated with a High Biological Fitness Cost
Source: Front Microbiol. 2017 Dec 5;8:2303. doi: 10.3389/fmicb.2017.02303 (PMC5723333; doi:10.3389/fmicb.2017.02303)

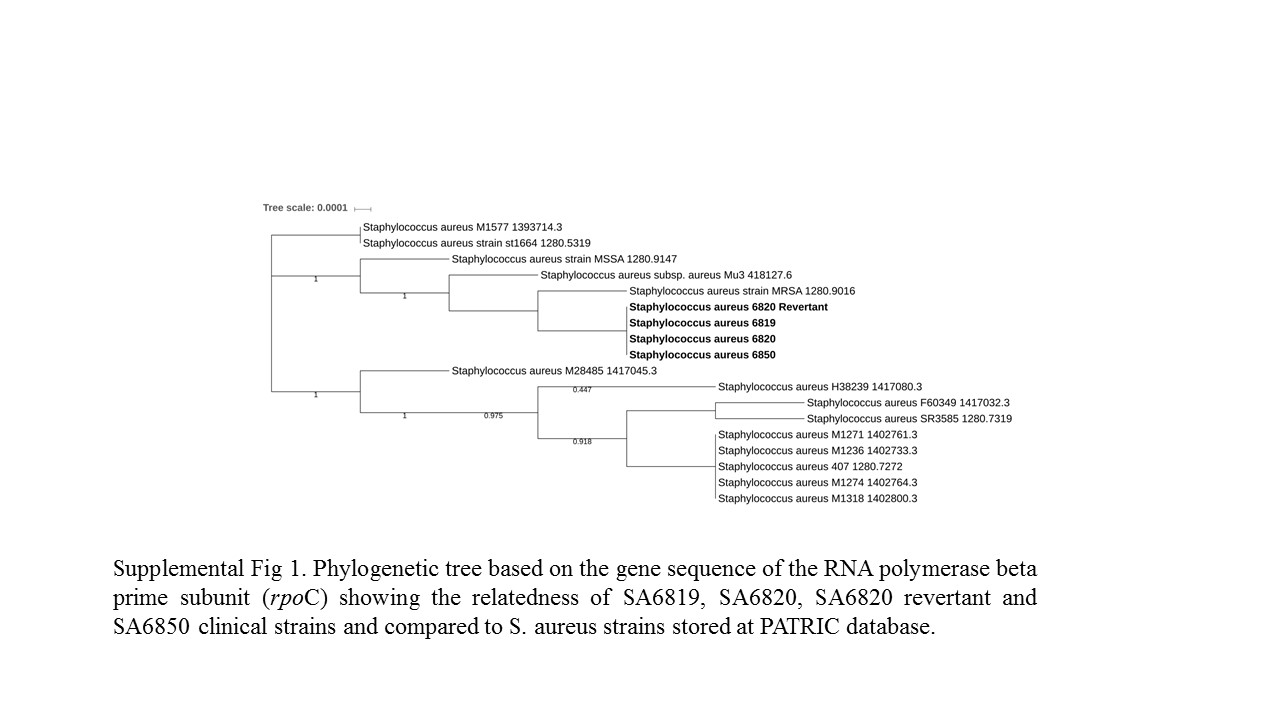

Supplement: Supplementary file 1 [file Image_1.jpg]
